# Supplementary material for: Effectiveness of mobile phone text message reminder interventions to improve adherence to antiretroviral therapy among adolescents living with HIV: A systematic review and meta-analysis
Source: PLoS One. 2021 Jul 22;16(7):e0254890. doi: 10.1371/journal.pone.0254890 (PMC8297901; doi:10.1371/journal.pone.0254890)
Supplement: S1 Appendix — (DOCX) [file pone.0254890.s001.docx]

**S1 Appendix – Search strategy**

Reporting of the search strategy

Host platform and databases searched:

1. PubMed sources: MEDLINE

2. EMBASE sources: EMBASE

3. WEB OF SCIENCE sources: Web of Science Core Collection

4. Cochrane Library sources: Cochrane reviews; Cochrane protocols; Trials; Editorials; Special Collections; Clinical Answers

5. CABI sources: Global Health

Date last search was run: 18 August 2020.

Years covered by search: All years available in each database were included in the search.

Language restriction: No language restriction.

Document restriction: No document types restriction.

Summary of the search strategy: The search strategy was developed to ensure a similar design/terminology across all host platforms of databases searched. The search strategy is divided into two groups of keywords:

#1: Text message reminders.

#2: HIV.

The two groups were combined with the following logic: #1 AND #2.

MEDLINE (PUBMED) (2160 results)

_ #1: (mobile phone* OR cell phone* OR text reminder* OR text messages* OR phone based*)

_ #2: (HIV OR AIDS OR ART OR Antiretroviral therap*)

COMPLETE STRATEGY (Copy and paste): (mobile phone* OR cell phone* OR text reminder* OR text messages* OR phone based*) AND (HIV OR AIDS OR ART OR Antiretroviral therap*)

EMBASE (72 results)

_ #1: (mobile phone* OR cell phone* OR text reminder* OR text messages* OR phone based*)

_ #2: (HIV OR AIDS OR ART OR Antiretroviral therap*)

COMPLETE STRATEGY (Copy and paste): (mobile phone* OR cell phone* OR text reminder* OR text messages* OR phone based*) AND (HIV OR AIDS OR ART OR Antiretroviral therap*)

WEB OF SCIENCE (202 results)

COMPLETE STRATEGY (Copy and paste): HIV mobile phone reminders

COCHRANE LIBRARY (102 results)

COMPLETE STRATEGY (Copy and paste): mobile phone reminders HIV

CABI (65 results)

COMPLETE STRATEGY (Copy and paste): mobile phone reminders HIV
