# Supplementary material for: Effectiveness of mobile phone text message reminder interventions to improve adherence to antiretroviral therapy among adolescents living with HIV: A systematic review and meta-analysis
Source: PLoS One. 2021 Jul 22;16(7):e0254890. doi: 10.1371/journal.pone.0254890 (PMC8297901; doi:10.1371/journal.pone.0254890)
Supplement: Attachment — Submitted filename: Respone to reviewers.docx [file pone.0254890.s004.docx]

**Response to reviewers**

We sincerely thank the reviewers for thorough reading of this manuscript and the editor for the opportunity to improve the manuscript. The suggestions and comments have been closely followed and revisions have been made accordingly. We have hopefully addressed the concerns to your satisfaction. Point-by-point responses to the comments are listed below. The revisions have been color tracked in the manuscript.

**Reviewer #1**: The authors stated that "the data are fully available without restriction" and "all relevant data are within the manuscript and its supporting files". I was not able to find any data files or analysis code. I'd be happy to complete a full review once the data and code are shared. I'm marking as "reject" at this point on the recommendation of the handling editor as a way to request access to these materials. This recommendation is not based on any assessment that the paper is unsuitable for publication.

Answer: We deeply regret the inconvenience to our reviewer. We have attached data files in the Appendix section: S3 Appendix – Data for meta-analysis. We are looking forward to receiving feedback from our reviewer.

**Reviewer #2:** To the authors:

This manuscript addresses an extremely important topic and I applaud the authors for undertaking this systematic review and meta-analysis to help us understand whether mobile phone SMS hold promise to support improved adherence in a known vulnerable population: adolescents with HIV. I enjoyed reading the manuscript. I found a few places that need clarification. With some careful editing, I believe it will be worthy of publication.

Specific Comments:

Abstract

1. In the Results section, 3rd sentence, the authors write, “… while no significant difference was found in the standard of care in the remaining two studies.” This is confusing. Do you mean that no difference was found between intervention and control (standard of care) groups? This should be clarified.

Answer: Thank you for pointing out this oversight. We understand that our statement was not clear at this point therefore, we have updated the sentence (Page number 2, line numbers 42 – 44) as follows: “Five studies showed a positive impact of text messaging in improving adherence, while no significant difference was found between the intervention and the control (standard of care) group in the remaining two studies.”

2. In the Conclusion and recommendation section, I think it would be good to highlight more prominently that this study could not confirm its motivating question: whether mobile phone text message interventions can improve adherence among HIV-positive adolescents. The meta-analysis failed to show a significant difference – it’s there in this section but it’s a bit buried between other points. This fundamental finding should be highlighted, I think.

Answer: As suggested by the reviewer, the Conclusion and recommendation section has been revised and it read as follows. (Page 2, line numbers 48 -54) “The meta-analysis did not show a statistically significant difference in improvement of ART adherence due to text message reminder intervention among adolescents living with HIV. The included studies were heterogeneous in the reported clinical outcomes where effectiveness of the intervention was identified in small studies which had a short follow-up period. Studies with a bigger sample size and a longer follow-up period are needed.”

Introduction

3. On page 3, second paragraph, there are some confusing numbers provided. In line 6 of the paragraph (lines 67-68) beginning with “With an emphasis on viral suppression…”, the sentence that says, “… of which 53% were aged 0-14 years and 68% aged 15 and older living with HIV” doesn’t make sense. Can you clarify these numbers?

Answer: Thank you for pointing out this oversight. The statement (Page number 4, line number 72-76) has been updated as follows: “At the end of 2019, 12.6 million (33% of people living with HIV) people did not have access to antiretroviral therapy (ART). An estimated 840 000 children did not have access to ART at the end of 2019, amounting to 47% of all children living with HIV.”

4. In the same paragraph, some of the references seem a bit old. For instance, the UNAIDS Global AIDS update is cited from 2017 – but the 2020 report is out, so I would recommend using the most recent numbers throughout this important background paragraph.

Answer: Thanks for this valuable input. Latest references have been updated on page 4, line numbers 74 and 75.

5. On page 4, in the first paragraph, top line of the page (line 75), the authors write, “Adherence to treatment, defined as how medication-taking behavior corresponds to the recommendations….” is not very helpful as an operational definition. What you mean is more like – “defined as taking HIV medications as prescribed, which includes taking them every day at roughly the same time, without missing doses.”

Answer: We have updated the definition of Adherence (page number 5, line numbers 82-85) as follows: “Adherence to treatment is defined as how patient’s medication-taking behaviour corresponds to their health care professional’s recommendations which include taking medications daily at about roughly the same time without missing doses.”

6. For the last sentence in the same paragraph (line 81), why is the Finitsis article on text message interventions the reference for a general statement on the importance of adherence in preventing progression to AIDS, morbidity, and death among PLHIV? I think it would be good to have a reference to a study whose purpose was to investigate the relationship between adherence and morbidity/death (such as Paterson et al (2000) and Arnsten et al (2001)).

Answer: We agree with the reviewer on this point. We have changed our reference to [Paterson et al (2000)](https://pubmed.ncbi.nlm.nih.gov/10877736/). (Page number 5, line number 90).

7. On the next page (5), in the last paragraph (line 109), the authors write about their study focusing on the effect of text message reminders to improve ART adherence – but don’t specify that they are also focusing on a specific population: adolescents. This is an important part of their study and should be included here.

Answer: Thanks for pointing this out. We have updated the statement (Page number 6, line number 118-119) as “In this study the effect of a text message reminder intervention on ART adherence in adolescents living with HIV is explored by performing a systematic review and a meta-analysis.”

Methods

8. In the section on Study inclusion criteria, the text on the adherence measure seems confusing and inaccurate. The text reads (in line 138) “Adherence to ART either as pill count or viral load as an outcome.” One problem with this as written is that, while both outcomes are important, and HIV viral load can be an indicator of past adherence, it is not a measure of adherence itself, strictly speaking. The second problem is that saying “…ART either as pill count or viral load” suggests that adherence will be measured as pill count only. Pill count has a specific meaning in measuring adherence, and it is distinct from such measures as self-report, MEMS, etc. and the latter are clearly included in the papers included in the review – so this is very confusing. I would recommend dropping the “pill count” altogether (as that is misleading) and then revising the line somewhat to read: “using either adherence to ART (measured in various ways) or HIV viral load as a study outcome”. In reading the manuscript, this captures what the authors actually did.

Answer: We agree with the reviewer’s suggestion and have updated the statement (Page number 8, line numbers 148-149) as “Using either adherence to ART (measured in different ways) or viral load as the study outcome.”

9. For study inclusion/exclusion generally, I would not use bullets. I would recommend writing out the specific criteria separated by “;” or I would put them in a simple table.

Answer: Based on reviewer’s suggestion we have edited the text. (Page numbers 7-8, line numbers 142-158)

Results

10. On page 9, in the first paragraph in the Study characteristics section, I would add a sentence clarifying where the five RCTs were done – how many in the USA how many in the four outside of the USA (e.g., in more resource-constrained environments)?

Answer: We have updated the text (Page number 10, line numbers 206-209) and it reads as follows: “Seven studies were included in the review; five RCTs (two in the USA and one each in Uganda, Nigeria, and Cameroon) and two cohort studies (one each in USA and Argentina) analysing the effect of mobile text reminders for improving adherence to ART among adolescents living with HIV were included”

11. On page 13, in the paragraph under the sub-heading “Summary of included studies,” the authors describe the adherence outcome and the fact that some studies used self-report and others used MEMS. This is a really important distinction because most ART adherence researchers accept that self-report is not a very accurate measure of adherence. So one recommendation is to specify clearly how many studies used self-report (Table 2 indicates that two did) here. Second, I think it will be important to acknowledge this limitation in the meta-analysis if studies that use self-reported adherence are included.
Answer: Thanks for this valuable insight. We have revised the text (page numbers 14-15, line numbers 233-237) as “Three studies (Hailey 2013, Abiodun 2019 and Ketchaji 2019) used self-reported data for outcome assessment, while two studies (Linnemayr 2014 and Mimiaga 2019) documented using a medication event monitoring system (MEMS) in addition to self-reported data.”.

As suggested by the reviewer we have updated our limitations of meta-analysis (page number 23, line numbers 342-345) as follows: “Moreover, five studies used self-reported data, which is considered a less sensitive measure of adherence than MEMS and tends to overestimate the impact. We think that inclusion of self-reported data in our meta-analysis may affect the validity of our findings and therefore is a limitation of this study.”

12. Table 2 – could you add the time frame for the adherence and viral load outcomes? It is important to know when those measures are taken. That is, for adherence, is it mean adherence over the intervention period, or in the last month? It’s hard to have a sense of the rigor of these studies without knowing when data were collected/analyzed for these outcomes.

Answer: We duly acknowledge reviewer’s views on the issue. We have changed the heading of the last column in Table 2 (page number 16) to “Adherence and/or viral load measurement^1”^. We have further added a note at the bottom of the table (page number 18) “Adherence and viral load measured at baseline and at the end of the follow-up period.” We hope that our modification can address the issue raised by the reviewer.

13. In Table 2, for the last study, were both MEMS and self-reported adherence used? If so, it might be better to use “and” there to be clear.

Answer: Thanks for the suggestion. We have updated Table 2 (page number 18) in this regard.

14. On page 18, line 250, the authors write, “Sub-group analysis of studies found no difference in mean ART adherence among adolescents who receive only text message (one way)….”. Do you mean that analysis found no significant effect on adherence from one-way text messaging?

Answer: At this point we wanted to say that the mean difference between one-way text messaging and control is zero. We have revised are statement (page number 19, line numbers 250-252) as “Sub-group analysis of studies found no difference in mean ART adherence (mean difference = 0.00) among adolescents who receive only text message (one way).” to clarify our viewpoint.

Discussion

15. I would add as a study limitation that adherence was measured in different ways, including the limitation related to self-reported adherence.

Answer: Thank you for this valuable insight. We have updated our limitations of meta-analysis (page number 23, line numbers 342-345) as follows: “Moreover, five studies used self-reported data, which is considered a less sensitive measure of adherence than MEMS and tends to overestimate the impact. We think that inclusion of self-reported data in our meta-analysis may affect the validity of our findings and therefore is a limitation of this study.”

16. Finally – while the writing is generally clear, there are a number of minor grammar errors that need correcting throughout the manuscript.

Answer: We have tried to correct grammatical errors in the manuscript. Additionally, we had our manuscript proofread by a native English speaker who has experience in academic writing.
