# Supplementary material for: Effectiveness of mobile phone text message reminder interventions to improve adherence to antiretroviral therapy among adolescents living with HIV: A systematic review and meta-analysis
Source: PLoS One. 2021 Jul 22;16(7):e0254890. doi: 10.1371/journal.pone.0254890 (PMC8297901; doi:10.1371/journal.pone.0254890)
Supplement: Attachment — Submitted filename: Response to reviewers.docx [file pone.0254890.s005.docx]

**Response to reviewers**

We sincerely thank the reviewers for the thorough reading of this manuscript and the editor for the opportunity to improve the manuscript. The suggestions and comments have been closely followed, and revisions have been made accordingly. We have hopefully addressed the concerns to your satisfaction. Point-by-point responses to the comments are listed below. In addition, the revisions have been colour tracked in the manuscript.

**Reviewer #1**: The authors conducted a meta-analysis of SMS reminder interventions on ART adherence among HIV-positive adolescents. My main concern is that the outcome is not clearly defined, and this made it hard for me to interpret the findings.

The authors write:

- "Adherence to treatment is defined as how patient’s medication-taking behaviour corresponds to their health care professional’s recommendations which include taking medications daily at about roughly the same time without missing doses."

- "adherence to ART (measured in different ways)"

- "The data used in the analysis were mean adherence rates"

- "Medication adherence was reported as a mean adherence rate, odds ratio, or using a visual analogue scale (VAS) score."

*Is adherence a binary outcome? If so, what made someone "adherent" in the primary studies?*

Response: Thank you for raising this important question. There was considerable heterogeneity in how adherence was reported in primary studies. Four out of seven primary studies reported adherence as a continuous variable where intervention was compared with a control group (Linnemayr et. al., Abiodun et al and Mimiaga et. al.) or a pre-and post-intervention adherence level was reported (Hailey et al). In these studies, the participants receiving the intervention were reported to be more (or less) adherent as compared to a control group and/or a pre-intervention state.

The study by Abiodun et. al., additionally reported a dichotomised variable based on a VAS score of >= 95%. Study by Ketchaji et. al., also reported a binary variable for adherence where criteria for dichotomisation was not reported.

The heterogeneity of the outcomes (continuous or binary) reported in the primary studies affected our choice of outcome measure for meta-analysis. Primary studies dichotomised mean adherence at different points. Therefore, we have updated our limitations as follows (Page 22, lines 345-347): “Finally, variation in reported outcome (continuous or binary) and their units posed a challenge to include most studies in the meta-analysis.”

*Is adherence defined as somone's adherent days / all days during each trial? Is it a pill count?*

Response: Thanks for highlighting this oversight. In the present study, adherence as an outcome variable is measured either as a pill count or MEMS counts (prescription drug bottle opening counts). In this regard, we have added the following sentences to our methodology (Page 8, lines 182-187) “Most studies included in this systematic review reported adherence to ART as a continuous outcome (mean adherence). Therefore, mean adherence was chosen as the outcome measure for quantitative analysis. For the meta-analysis, adherence is defined as how a participants’ pill count or MEMS data corresponds to their doctor’s prescription regimen during the period of trial. Mean adherence is a continuous variable that can take a value from 0-1, where 0 represents nil adherence, and 1 represent perfect adherence.”.

*I searched a bit and confirmed my hunch that many (most?) meta-analyses of medication adherence studies report the effect sizes as odds ratios. It's possible that I'm out of touch on this, but I would benefit from an explanation from the authors about why mean differences of rates makes the most sense for this meta-analysis.*

Response: We thank the reviewer for asking this important question. We agree with the reviewer that most studies used the odds ratio. Mean adherence is an absolute measure of effect as opposed to odds ratio, which is a relative measure of effect. The outcome variable needs to be binary to investigate the odds ratio. If the outcome measure for the analysis is a continuous variable, then it needs to be dichotomised at a specific point. For ART adherence, mean adherence rate is dichotomised at the point where viral suppression is assumed to have achieved.

In the current study, we undertook a comprehensive review of peer-reviewed and grey literature. We decided that having an absolute measure of effect (mean difference) in our meta-analysis will be more suitable due to the following reasons:

1. Using mean adherence rates allowed us to include more studies in the meta-analysis.
2. We were interested in examining whether text message reminder can improve adherence. Mean difference is a commonly used measure of effect in meta-analysis.
3. Mean difference evaluates a continuous variable (mean adherence) and, therefore, is not based on the assumption that viral suppression is achieved at a specific level of adherence.

In this regard, we have updated our methods section as follows (Page 8, lines 182-184) "Most included studies in this systematic review reported adherence to ART as a continuous outcome (mean adherence). Therefore, mean adherence was chosen as the outcome measure for quantitative analysis.". We further updated our limitations as (Page 22, lines 347-350) "Odds ratio is a more common outcome measure for adherence in a meta-analysis that evaluates a binary outcome. However, in the current study, the included studies dichotomised adherence at different points that posed a challenge to have odds ratio as an outcome measure."

My other comments are minor and should not stand in the way of publication if that is the editor's decision:

- *Unclear why cohort studies were included*

Response: We thank the reviewer for raising this question. The current study aimed to gather evidence of the effectiveness of text message reminder intervention. In addition to randomised studies, a cohort (non-randomised) study design can demonstrate the effectiveness of interventions. Therefore, cohort studies were made a part of the inclusion criteria. The inclusion criteria were decided a priori, given the limited number of randomised trials anticipated in this area.

- *Unclear why interventions were limited to SMS (the field has expanded so much since SMS was dominant, e.g., WhatsApp)*

Response: We thank the reviewer for this comment. The accessibility of smartphones (having WhatsApp) has been increasing worldwide, especially in high and upper-middle income countries but is currently lower in low and low-middle income countries. So, we first wanted to determine if this primary digital function, SMS, which is available almost everywhere, could have any beneficial impact. In the "Introduction", we have the following statement (Page 5, lines 120-122) "A text message can be received on a mobile phone with basic features. This intervention curtails stigma and can be rolled out in a resource-limited setting.".

- *The introduction does not mention anything about the evidence in favor of SMS reminders for adherence among adults living with HIV (does appear in the discussion)*

Response: We thank the reviewer for this question. We would like to mention that in the “Introduction” section, we have the following statement to inform on the evidence in favour of SMS reminder for adherence among adults living with HIV (Page 5-6, lines 121-124) “Previous studies have shown the effectiveness of mHealth interventions in improving treatment adherence among adults living with HIV (35-38), and the evidence is emerging in the adolescent age group.”. Four peer-reviewed references support our statement. We acknowledge that we have a more detailed narration on these studies in our "Discussion" section, where we have compared our findings with previous studies in this area.

- *The overall reporting of the methods and results could be improved*

Response: We thank the reviewer for this helpful suggestion. Based on the reviewers' comments and suggestions, we have tried to improve our methods and results section and tracked changes are made in the manuscript in this regard.

*- Why is the age range of adolescents limited to 19? The definition varies across settings. I think more studies could have been included with a broader definition.*

Response: We agree more studies could have been included if the age criteria was expanded. However, we utilised the definition of adolescents age group as recommended by the World Health Organisation. We aimed to focus our evidence search on the adolescent age group (defined by WHO) for two reasons. Firstly, we found out some unique challenges related to ART adherence and retention care in this age group. Secondly, we wanted to focus on identifying adolescent-friendly interventions that can improve adherence and retention in care in this unique age group.

**Reviewer #2**: In this resubmitted version of the manuscript, the authors addressed the points in my original review. Some areas of the revised text are still a bit confusing, however. With a few relatively minor edits, I believe the manuscript will be worthy of publication and make an important contribution to the field.

Specific Comments:

Abstract

1. *The authors clarified the main study finding, that the meta-analysis failed to show a significant difference of mobile phone text message reminders, but the text is a bit confusing (Page 2, line numbers 48-49). I would reorganize the first sentence slightly for clarity as follows: “**The meta-analysis of text message reminder interventions did not show a statistically significant difference in improvement of ART adherence among adolescents living with HIV.”*

Response: Thanks for the suggestion. We have rewritten the statement as advised (Page 2, line number 48-50).

Introduction

2. *With reference to the edits made to define adherence (page number 4, line numbers 78-81), the authors made helpful edits, but “about” should be removed in the following sentence to avoid redundancy: “Adherence to treatment is defined as how patient’s medication-taking behaviour corresponds to their health care professional’s recommendations which include taking medications daily at [about—remove] roughly the same time without missing doses.”*

Response: Thanks for the suggestion, we have rewritten the statement as advised (Page 4, line number 78-81).

Discussion

3. *Adding that use of self-report as an adherence measure by some of the included studies in the meta-analysis is a study limitation is helpful (page 22, lines 342-344). However, some of that text is not quite accurate, as use of self-report overstates adherence, not impact. Rather than writing “…tends to overestimate the impact” I suggest that the authors say “…tends to overestimate adherence.” Also, it would be good to introduce that paragraph on limitations with something like: “We also acknowledge a number of study limitations…..”*

Response: Thanks for this valuable suggestion. We have added a sentence (Page 22, lines 339) “We also acknowledge some limitations in our study.”

We have rewritten another statement in the paragraph (Page 22, lines 342-344) “Moreover, five studies used self-reported data, which is considered a less sensitive measure of adherence than MEMS and tends to overestimate the adherence”.

4. *Finally –* *there are still a lot of minor grammar errors and typos that need correcting in the manuscript. I'm sorry I don't have time to point out all the places that need editing. However, here are two examples, both from the Methods section of the Abstract. First, the second sentence beginning with “Literature search was done…” should read, “A literature search was done…” Second, the next to last sentence which ends with, “…computed using a random effect model” should read “…computed using a random effects model." The manuscript needs careful editing by a native English speaker to remove all the errors and typos before it can be published.*

Response: Thanks for pointing this out. A native English speaker has reviewed the manuscript. We have made tracked changes throughout the manuscript.
