# Supplementary material for: Effectiveness of mobile phone text message reminder interventions to improve adherence to antiretroviral therapy among adolescents living with HIV: A systematic review and meta-analysis
Source: PLoS One. 2021 Jul 22;16(7):e0254890. doi: 10.1371/journal.pone.0254890 (PMC8297901; doi:10.1371/journal.pone.0254890)
Supplement: Attachment — Submitted filename: Response to reviewers.docx [file pone.0254890.s006.docx]

**Response to reviewers**

We sincerely thank the reviewers for the thorough reading of this manuscript and the editor for the opportunity to improve the manuscript. The suggestions and comments have been closely followed, and revisions have been made accordingly. We have hopefully addressed the concerns to your satisfaction. Point-by-point responses to the comments are listed below. In addition, the revisions have been colour tracked in the manuscript.

Reviewer #2: Thank you for addressing the comments of reviewers of the previous submission. It is much improved since the previous version. I have two remaining issues that I hope the authors will address.

1. *On page 4, line 83, the authors write that adherence >95% is necessary for optimal viral suppression, and cite an article from 2000. ART regimens have improved substantially since 2000 and the precise level of “necessary” adherence for viral suppression is unclear (and is somewhat of a controversial subject). Numerous articles since 2000 have highlighted this point. Please see, for example:

**Bangsberg DR. Less than 95% adherence to nonnucleoside reverse-transcriptase inhibitor therapy can lead to viral suppression. Clin Infect Dis. Oct 1 2006; 43(7): 939-41.

**Parienti JJ, Das-Douglas M, Massari V, Guzman D, Deeks SG, Verdon R, Bangsberg DR. Not all missed doses are the same: sustained NNRTI treatment interruptions predict HIV rebound at low-to-moderate adherence levels. PLoS One. 2008; 3(7): e2783.

**Haberer JE, Sabin L, Amico KR, Orrell C, Galarraga O, Tsai AC, et al. Improving antiretroviral therapy adherence in resource-limited settings at scale: a discussion of interventions and recommendations. J Int AIDS Soc. 2017; 20(1): 21371.*

Response: We would like to thank our reviewer for this valuable insight. We have updated our text and included relevant references. The following statements have been included in the manuscript on page 4 Line 84-86 – “However, recent studies suggest that even moderate adherence to potent ART regimens can achieve viral suppression (Bangsberg, 2006). Parienti et al. point to sustained treatment interruptions leading to viral rebound rather than interspersed missed doses (Parienti, 2006).”.

2. *The authors state that a native English speaker has reviewed the manuscript, but it still has numerous typos and sentences with incorrect grammar. Two examples are from the first page of the main manuscript: 1) Line 60: the sentence now beginning with “Latest estimates suggest…” should start with “The” so that the sentence reads, “The latest estimates suggest….”; 2) Lines 69-70: the sentence that reads “At the end of 2019, 12.6 million (33% of people living with HIV) people did not have…” should be revised to read: “At the end of 2019, 12.6 million people (33% of people living with HIV) did not have….” I suggest that the authors can do further proofreading and editing so that the manuscript is error free.*

Response: We appreciate our reviewer’s concern. To ensure that our manuscript is error free, it has been reviewed again by a native English speaker. The grammatical corrections are shown as tracked changes throughout the manuscript.

**Changes to the reference List**

We had one retracted refence in our list that was replaced with a new reference. Furthermore, we identified that four of references are available at a different web location and therefore, they were updated. Lastly, we included 2 new references as per our reviewer’s suggestion above.

*Retracted reference* – (6) United Nations Children's Fund. Early and late adolescence: UNICEF; 2011 [Available from: <https://www.unicef.org/sowc2011/pdfs/Early-and-late-adolescence.pdf>].

*New reference* – (6) World Health Organization. Stages of Adolescent Development 2010 [Available from: <https://apps.who.int/adolescent/second-decade/section/section_2/level2_2.php>].

*Old reference* – (10) World Health Organization. Adherence to long-term therapies: evidence for action: World Health Organization; 2003 [Available from: <https://www.who.int/chp/knowledge/publications/adherence_report/en/>].

*Updated reference* – (10) World Health Organization. Adherence to long-term therapies: evidence for action Switzerland2003 [Available from: <https://apps.who.int/iris/bitstream/handle/10665/42682/9241545992.pdf>].

*Old reference* – (19) World Health Organization. Chapter 9: Guidance on operations and service delivery: WHO; 2013 [Available from: <https://www.who.int/hiv/pub/guidelines/arv2013/operational/adherence/en/>].

*Updated reference* – (19) World Health Organization. Chapter 9: Guidance on operations and service delivery: WHO; 2013 [Available from: <https://www.who.int/publications/i/item/9789241505727>].

*Old reference* – (23) World Health Organization. HIV and adolescents: guidance for HIV testing and counselling and care for adolescents living with HIV: recommendations for a public health approach and considerations for policy-makers and managers 2013 [Available from: <https://www.who.int/hiv/pub/guidelines/adolescents/en/>].

*Updated reference* – (23) World Health Organization. HIV and adolescents: guidance for HIV testing and counselling and care for adolescents living with HIV: recommendations for a public health approach and considerations for policy-makers and managers 2013 [Available from: <https://www.who.int/publications/i/item/9789241506168>].

*Old reference* – (26) World Health Organization. Global diffusion of eHealth: Making universal health coverage achievable. Report of the third global survey on eHealth: WHO; 2016 [Available from: <https://www.who.int/goe/publications/global_diffusion/en/>].

*Updated reference* – (26) World Health Organization. Global diffusion of eHealth: Making universal health coverage achievable. Report of the third global survey on eHealth: WHO; 2016 [Available from: <https://www.who.int/publications/i/item/9789241511780>].

*Added reference* – (14) Bangsberg D. R. Less Than 95% Adherence to Nonnucleoside Reverse-Transcriptase Inhibitor Therapy Can Lead to Viral Suppression. Clinical Infectious Diseases. 2006;43(7):939-41. doi 10.1086/507526

*Added reference* – (15) Parienti J. J., Das-Douglas M., Massari V., Guzman D., Deeks S. G., Verdon R. et al. Not all missed doses are the same: sustained NNRTI treatment interruptions predict HIV rebound at low-to-moderate adherence levels. PLoS One. 2008;3(7):e2783. doi 10.1371/journal.pone.0002783
